# Cluster randomized multilevel intervention for promoting physical activity in rural communities

**DOI:** 10.3389/fpubh.2025.1577056

**Published:** 2025-08-29

**Authors:** Alan M. Beck, Natalicio Serrano, Dixie Duncan, Amy A. Eyler, Amanda Gilbert, Fatemeh Naghiloo, Rodrigo Reis, Rachel G. Tabak, Ross C. Brownson

**Affiliations:** ^1^Prevention Research Center, School of Public Health, Washington University in St. Louis, St. Louis, MO, United States; ^2^Gillings School of Global Public Health, University of North Carolina, Chapel Hill, NC, United States; ^3^Children’s National Hospital, Washington, DC, United States; ^4^Alvin J. Siteman Cancer Center, Washington University School of Medicine, St. Louis, MO, United States

**Keywords:** rural health, prevention, physical activity, multilevel intervention, text messaging

## Abstract

**Introduction:**

Rural areas of the United States have lower levels of physical activity (PA) as compared to their urban and suburban counterparts, leading to higher levels of chronic diseases. The objective of the study was to increase PA in rural communities via a multilevel intervention.

**Methods:**

We implemented a cluster randomized controlled trial of a community-based multilevel PA intervention between 2019 and 2022 in 14 rural communities. The intervention consisted of (1) text messaging (i.e., individual level), (2) encouragement of walking groups (i.e., interpersonal level), and (3) community-based marketing (i.e., community level).

**Results:**

Participants meeting the 2018 PA guidelines for aerobic PA increased among the intervention group, with no differences in recreational, occupational, transportation, or total PA.

**Discussion:**

In this multilevel study the intervention is associated with meeting PA guidelines. The complexities of the impact of COVID-19 make it hard to disentangle the effects of the intervention on PA and other outcomes. Though the main tenets of the multilevel intervention had to be altered due to the pandemic, there was value in text messaging in rural areas to promote PA. Future studies should consider incorporating text messages into rural areas as a component of a PA promotion campaign.

**Clinical trial registration:**

Clinicaltrials.gov, identifier NCT03683173.

## 1 Introduction

While there are myriad risk factors for chronic diseases, physical activity (PA) is a low-risk, high-benefit health-promoting behavior lowering the risk of many chronic diseases (1). PA occurs through leisure (e.g., walking, running, cycling), transportation (e.g., walking to work, cycling to school), and/or occupational (e.g., construction, farming, factory work) activity with multiple aspects of a person (e.g., personal behavior, neighborhood environment, policy) influencing behavior change (2). Many Americans do not meet PA recommendations (1); however, rural Americans are the least likely to meet PA recommendations, which puts them at an elevated risk for chronic disease (3). Low levels of PA in rural communities are likely due to many contextual elements affecting these interactions (2). As PA behaviors are driven by an individual’s interaction within their environment (geographical, social, and cultural), the coalescence of barriers at multiple levels creates an imperative to intervene to promote the health benefits of PA.

Rural communities lack evidence concerning what interventions lead to increased PA. Of the 205 programs in the US National Cancer Institute’s compendium of evidence-based interventions, only 7 (i.e., 3.4%) focus on rural PA promotion (4). Even smaller numbers involve multilevel intervention approaches. One multilevel study found small increases in PA among a sample of nearly 200 rural women (5). Some studies have used walking groups as a potential option for increasing PA among rural communities (6). Finally, there is promise for cost-effective text messaging to increase PA among rural adults (7).

This study focused on rural southeast Missouri where walking trails (i.e., trails) have been built in the region to promote PA (8). A pilot study revealed trails are underused, and adults reporting regular use of trails were more likely to meet PA recommendations (9). Therefore, changing the built environment is likely necessary, but not a sufficient approach to increasing PA (10). The study aimed to test the effects of a multilevel intervention on increasing PA in rural communities. We hypothesized the multilevel intervention would increase PA among the participants.

## 2 Methods

### 2.1 Study design

*Heartland Moves* was a cluster randomized controlled trial, with randomization at the community level (11). The 14 communities were paired based on population size, socioeconomic status (i.e., percent below poverty line), and diversity (i.e., percent non-white). Rurality was defined using the Rural–Urban Continuum Codes (RUCC) where one equates to the most urban, and nine equates to the most rural (12). Communities were between RUCC 3–9. One community was delineated as a metro county (i.e., RUCC 3), but its population and demographics were comparable to one community in RUCC 4 delineated as non-metropolitan with an urban population of 20,000 or more, adjacent to a metro area. The remaining communities were RUCC 5 (one community), RUCC 6 (two communities), RUCC 7 (5 communities), RUCC 8 one community, and RUCC 9 (three communities). The population of included communities ranged from 1,800 to 16,000. Adult participants were recruited via community outreach, events, and address-based sampling. Inclusion criteria were the ability to participate in PA, read the English language, and aged 18 years or older. Exclusion criteria were cognitive impairment, inability to be active, or not residing in one of the intervention or control communities. Baseline data were collected by telephone between June 2019 and September 2020, and follow-up data were collected between December 2021 and March 2022, on a rolling basis (to correspond to the pairing of intervention and control communities). Before data collection, informed consent was obtained from all participants. This study was approved by the Institutional Review Board of the sponsoring institution (#201809089) and registered with clinical trials (NCT03683173).

### 2.2 Randomization and power analysis

A list of potential participant communities was compiled based on prior work in the area – the only criterion considered was at least one serviceable trail. Communities were paired based on similar population sizes. Sixty-four possible permutations were run based on population, percent below poverty, and percent non-white to determine the best fit for randomization to the intervention or control condition. The paired communities were comparable in their demographic makeup. The intervention was implemented, and follow-up data were collected roughly one year from the start date.

With an average of 48 study subjects in each community, the study has a power greater than 90% to detect a difference between the control and intervention conditions. To achieve power of 90% to detect an intervention effect equivalent to a 16% increase in weekly MVPA minutes, using a two-level mixed effects model, we were required to enroll 1,200 participants at baseline, which was achieved.

### 2.3 Community participation

The *Heartland Moves* program was a multilevel intervention focusing on the individual, interpersonal, and community levels of the Social Ecological Model (2). Each intervention community was engaged to ensure that community representatives co-designed activities. Few communities had existing health coalitions, while other intervention sites developed multi-sector coalitions (e.g., city officials, nonprofit organizations, community champions, local businesses). Coalitions provided input on all levels of intervention design, including feedback and input on community specific text messages, walking group structure, leader training, and events. While activities were not implemented fully due to COVID-19, some coalitions sustained efforts post-intervention (walking groups, events, marketing local trails).

### 2.4 Individual-level intervention

Two short-message services (SMS; text messages) were used for intervention communities: (1) an exercise program, and (2) a community-specific outreach program. The SMS intervention was important for a few reasons. Previous research provides evidence for the potential effectiveness of SMS in promoting PA (13). For example, one systematic review and meta-analysis found SMS interventions led to higher rates of objectively measured PA (14). SMS interventions are flexible due to the ubiquity of cellular phones with text messaging across the United States, a cost effective and convenient way to reach populations, particularly underserved populations such as rural communities (15); however, utilizing SMS to promote PA in rural populations has limited evidence. Finally, the SMS intervention was implemented as intended during COVID-19, while supporting COVID-19 adaptations to the interpersonal and community-level interventions.

The SMS exercise program, developed by CareMessage, has been effectively used across differing populations for chronic disease prevention and management (16). The program is 24-weeks in duration, with three contact points per week. Messages focused on education related to benefits and types of exercise options, for example, *“Exercise does not have to happen in a gym. The goal is to get your body moving. Find something you enjoy such as dancing or walking with a good friend.”* There were also built in evaluative messages related to the program content and behavior, for example *“How confident are you that you can stick to your exercise program after a long, tiring day? (A) Very confident,’ (B) Confident, (C) Not confident, (D) Not at all confident.”*

The research team and community partners also developed a community-level SMS outreach program encouraging PA by promoting local PA resources (e.g., trails) and suggesting PA opportunities supporting physical and mental health, and social connection. Highlighting the physical health, mental health, and social connection benefits of PA provided education on the empirical benefits of being active and enhanced motivation (13). Messages were tailored to each community and focused on different benefits of PA and community-specific resources for example, “Hi from Heartland Moves. The ***** **** center has a 1.33 mile loop trail, 5 fitness stations, and 2 free tennis courts. Go enjoy!.” The personalized messages were sent twice per week for 12 weeks. When intervention participants consented to participate, they were asked if they wanted to receive SMS; thus, not all participants received text messages.

### 2.5 Interpersonal-level intervention

Walking groups were planned for each intervention community whereby a walking-group leader (a local volunteer) tracked the frequency, duration, and attendance of walking group meetings. However, adaptations were necessary due to COVID-19. Research staff contacted local community leaders to determine the most appropriate adaptation. It was noted walking groups were already forming (e.g., families, co-workers), therefore, we encouraged using walking groups via our outreach SMS and walking group guides mailed to intervention participants (*n* = 612). SMS messaging focused on walking with a friend or family member regularly, while the guide centered on three components: (1) how to start and maintain a walking group, (2) local places to partake in walking (e.g., trails), and (3) our contact information for any questions or concerns.

### 2.6 Community-level intervention

Events at local trails were planned at the community level; however, adaptations had to be made due to COVID-19 gathering restrictions. We created community mailers featuring “local celebrities” who were avid trail users in the community. We interviewed a local trail-user in each intervention community to ask them about their use of the trails, why being active is important, and what would encourage their neighbors to be active. We also included local trails and our contact information (*n* = 759 mailers). In one community, situated between two bisecting interstate highways, we created two billboards, as community members reported receiving health information that way. Research and communication staff used baseline data to determine appropriate messaging. *“Being active is family time”* depicted a local father and daughter using the trail, and *“Walk your way to better health”* depicted a group of older adults walking in a group. The messaging was tailored to community values including themes of family time for younger adults with children and a health and social support message for older adults. We used community members in the photos and partnered with community health nonprofits on the signage.

## 3 Data collection

### 3.1 Physical activity

Physical activity was measured via the Global Physical Activity Questionnaire (GPAQ) (17). The GPAQ is a subjective measure of recreational, transportation, occupational, and total PA, and has been adopted as a valid and feasible method for assessing change in PA (18). The GPAQ is reported to have moderate validity compared to accelerometry regarding moderate to vigorous PA (17). Standard procedures for calculating domain-specific outcomes from GPAQ were used for moderate to vigorous PA. Outcomes are presented as a dichotomous measure of whether recommended guidelines of PA were met (i.e., at least 150 min of moderate to vigorous PA calculated with one vigorous PA minute equating to two moderate PA minutes) and as continuous measures of each domain. The study relied on self-reported PA measures as participants were wary of receiving devices during the COVID-19 pandemic.

### 3.2 Intervention component

To examine the dose–response of the SMS intervention component, messages to participants were characterized as community outreach messages (i.e., messages personalized for communities), exercise messages (i.e., 24-week set exercise program), and potential responses to messaging. The number of messages received or responded to for each category was broken down as none, less than 50%, or more than or equal to 50% of all possible messages, with cut-points lining up with the median number of responses or messages received and being more practically relevant to communities. In total, 451 participants chose to receive messages. Participants were allowed to stop the SMS at any time during the study. Additionally, we collected survey data on any participation in walking groups, and exposure or knowledge of physical activity programming and/or activities in participant communities.

### 3.3 Statistical analysis

We obtained descriptive statistics (i.e., means and frequencies) on continuous self-report weekly combined moderate-vigorous PA measures (total, occupational, transportation, and recreational) including PA guideline adherence, baseline demographics, and dose–response measures on the SMS component of the intervention. We fitted generalized linear mixed-effects models to evaluate the intervention effects on PA outcomes. Models were adjusted for the clustering effect of community and controlled for the baseline measure of the outcome, age, gender, and education (19). A negative binomial distribution was used to account for the skewed distribution of the continuous PA measures. Negative binomial models are widely applied to continuous PA measures and are relevant to our sample as weekly minutes of PA are discrete, finite, non-negative, and skewed towards lower values (19). Adjusted prevalence estimates presented as means provide the number of minutes participants spend in PA by domain, as well as overall at follow-up controlling for the baseline measure of the outcome. Additionally, effect sizes are presented using Cohen’s f-squared (20). Further, to assess significance in meeting PA guidelines, walking group participation, and exposure to knowledge of PA programming and/or activities by group, a multilevel mixed logistic model was used to account for the clustering effect of community controlling for the baseline measure of the outcome, age, gender, and education. The adjusted odds ratio represents the odds of each outcome at follow-up for intervention participants compared to those in the control condition and adjusting for the clustering effect of communities and baseline covariates. Additionally, with the sample of intervention participants who completed follow-up measures, generalized linear mixed models were used to examine the dose–response effects of SMS components on the PA measures, adjusting for the clustering effect of community and controlling for the baseline measures of the outcome, age, gender, and education. Given the outcome is a count statistic and the measure followed a skewed distribution toward lower values, a negative binomial error distribution was used. All analyses were conducted on STATA S. E. Version 17.0.

## 4 Results

### 4.1 General characteristics of participants

Overall, 492 participants completed the follow-up data collection – 251 from the control condition and 241 from the intervention – which is a retention rate of 40% (1,241 at baseline [Table 1]). The final analytic sample consisted primarily of white women (68%), with a high school education or less (71.7%), and a household income of less than $50,000 (57%) (Table 1) – nearly half of the participants met PA guidelines.

**Table 1:** Baseline characteristics of participants by study condition and overall.

	Intervention (*n* = 241)	Control (*n* = 251)	Overall (*n* = 492)
Demographics	Mean (SD) or *n* (%)	Mean (SD) or *n* (%)	Mean (SD) or *n* (%)
Age, years	57.8 (16.3)	58.3 (15.8)	58.0 (16.0)
Gender (Woman)	162 (67.2%)	172 (68.5%)	334 (67.9%)
Education (More than high school completed)	**75 (31.1%)**	**64 (25.5%)**	**139 (28.3%)**
Annual household income < $50,000	140 (58.1%)	140 (55.7%)	280 (57.0%)
Race (non-White)	41 (17.0%)	38 (15.1%)	79 (16.1%)
Self-reported PA, min/week			
Recreational MVPA	140.9 (324.4)	100.2 (200.4)	120.1 (268.7)
Median (IQR)	0 (0.180)	0 (0.120)	0 (0.150)
Occupational MVPA	435.95 (1051.3)	360.92 (803.3)	398.0 (933.9)
Median (IQR)	0 (0.180)	0 (0.300)	0 (0.240)
Transportation-related MVPA	69.6 (240.4)	48.1 (156.1)	58.7 (202.0)
Median (IQR)	0 (0.15)	0 (0.0)	0 (0,0)
Total MVPA	640.3 (1190.5)	503.2 (854.6)	570.2 (1033.7)
Median (IQR)	140 (0, 600)	180 (0.585)	150 (0.600)
Meets MVPA recommendations	123 (51.0%)	133 (53.0%)	256 (52.0%)
Text messaging intervention characteristics			
Exercise messages	49 (39)	-	-
Outreach messages	17 (11)	-	-
Responses	6 (7)	-	-

PA: physical activity; MVPA, moderate-to- vigorous physical activity; PA, physical activity; SD, standard deviation.

Bold indicates a significant difference in group status (i.e., Intervention vs. Control).

### 4.2 Main outcomes

Although participants in the intervention arm had higher minutes of all continuous PA measures, the intervention had no statistically significant net effect at follow-up on continuous PA measures when compared to those in the control arm [i.e., total, transportation-related, recreational, and occupational weekly minutes of moderate-to-vigorous PA (Table 2)]. However, the odds of meeting PA guidelines (aerobic) were higher at follow-up among the intervention (OR = 1.99, 95% CI = 1.01, 3.93). When examining the unadjusted percentage of participants who met guidelines, 40.6 and 49.0% met guidelines in the control and intervention arms, respectively. When exploring socio-demographic and outcome differences in those who completed follow up and those who did not, participants who completed follow up had significantly lower education levels and more minutes of recreational PA, thus had a higher percentage of participants meeting recommended guidelines (Table 3). Though there were significant retention differences between groups with regard to education, there were no differences in PA measures between the groups, though it is important to note intervention effects may be more relevant to those who already have higher levels of PA.

**Table 2:** Mixed effects models to evaluate a multilevel physical activity intervention 12 months post-baseline (*N* = 492).

Intervention vs. control^a^	Odds Ratio	95% CI	*p*-value
Meets MVPA recommendations	**1.99**	**1.01–3.93**	**0.045**
Walking group participation	0.35	0.11–1.19	0.094
Community PA knowledge exposure	1.47	0.77–2.79	0.243

Condition	Meets MVPA recommendations	Walking group participation	Community PA knowledge exposure
Intervention, *n* (%)	118 (49.0%)	22 (16.8%)	112 (48.5%)
Control, *n* (%)	102 (40.6%)	23 (14.2%)	85 (36.5%)

	Intervention		Control		Difference (Inter-control)		
	Adj. Mean	SE	Adj. Mean	SE	Diff. in Adj. Means	*P*-value	Effect size^d^
Recreational MVPA minutes^b,c^	148.9	27.5	91.1	16.1	57.8	0.233	0.39
Occupational MVPA minutes^b,c^	406.9	96.1	392.8	93.3	14.2	0.897	0.05
Transportation-related MVPA minutes^b,c^	67.4	17.7	51.7	13.1	15.7	0.728	0.17
Total MVPA minutes^b,c^	612.6	77.5	519.7	64.0	92.9	0.563	0.13

MVPA, moderate-to-vigorous physical activity.

^a^Binomial error distribution (Logistic model).

^b^Generalized linear mixed models were used to adjust for the clustering effects of community.

^c^All analyses utilized a negative binomial error distribution and adjusted for the baseline measure of the outcome, age, gender, and education.

^d^Effect size is an estimate of Cohen’s *f*-squared.

^e^Bold indicates significant differences found between groups.

**Table 3:** Baseline characteristics of participants by completers and non-completers of follow-up.

Demographics	Completers (*n* = 492)	Non-completers (*n* = 743)	Overall (*N* = 1,241)
	Mean (SD) or %	Mean (SD) or %	Mean (SD) or %
Age, years	58.0 (16.0)	56.3 (17.1)	57.0 (16.7)
Gender (Woman)	67.9%	67.9%	67.9%
Education (More than high school completed)	**28.3%**	**37.2%**	**33.5%**
Annual household income < $50,000	57.0%	60.8%	59.2%
Race (non-White)	16.1%	16.1%	16.1%
Self-reported PA, min/week			
Recreational MVPA	**120.1 (268.7)**	**49.8 (149.1)**	**78.0 (208.4)**
Occupational MVPA	498.0 (933.9)	477.9 (1080.6)	445.8 (1024.6)
Transportation-related MVPA	58.7 (202.0)	55.0 (215.1)	56.4 (209.9)
Total MVPA	570.2 (1033.7)	588.8 (1192.8)	581.4 (1131.1)
Meets MVPA recommendations	**52.0%**	**45.6%**	**48.2%**

*Heartland Moves.*

PA, physical activity; BMI, body mass index; MVPA, moderate-to- vigorous physical activity; PA, physical activity; SD, standard deviation.

Bold indicates a significant difference in group status (i.e., Completers vs. non-Completers).

### 4.3 Dose–response

Among those who received the intervention, the SMS dose–response analyses showed participants who received half or more of the exercise messaging had significantly higher self-reported total weekly moderate-to-vigorous PA minutes than those who received no exercise messaging (Table 4). There was no significant dose–response relationship between the number of outreach messages or responses and total weekly moderate-to-vigorous PA minutes.

**Table 4:** Dose–response for text messaging component of intervention condition on total weekly MVPA 12 months post-baseline (*N* = 241).

Text messaging characteristic	Number of messages received or responses					
	None (ref)		<50%		≥50%	
	Adj. Mean	SE	Adj. Mean	SE	Adj. Mean	SE
Number of exercise messages	376.1	132.1	629.3	159.8	**818.0**	**148.4**
Number of outreach messages	399.6	131.3	551.1	191.4	791.9	127.0
Number of responses	394.4	121.6	835.7	295.1	615.7	265.5

^a^Mixed effects or generalized linear mixed models were used to adjust for the clustering effects of communities and to account for repeated measures. All analyses utilized a negative binomial error distribution and were adjusted for the baseline measure of the outcome, age, gender and education.

^b^Bold indicates significance at the 0.05 level.

## 5 Discussion

This study describes the results of *Heartland Moves,* a multilevel PA study in rural southeast Missouri. This study aimed to increase PA among adult residents via SMS, walking group participation, and a community communications plan. We found mixed results with self-reported data showing no effect on minutes of activity but a positive effect on meeting the guidelines for aerobic PA. This may be a product of control participants having a lower percentage of meeting guidelines compared to baseline, and those participating in the intervention staying at about the same percentage. Additionally, this study took place during the height of the COVID-19 pandemic, when PA levels were globally reduced (21). Baseline data noted over half of the sample met PA guidelines, which aligns with national datasets (22, 23). The amalgamation of baseline PA, and the intervention during COVID-19 impacted our findings.

### 5.1 SMS intervention

Importantly, the SMS intervention was not altered due to COVID-19 and retained fidelity to the study protocol. Delivering the SMS component of the multilevel intervention during COVID-19 provides evidence for the reach, feasibility, and sustainability of SMS interventions in rural communities. Our ability to deliver the SMS intervention supports prior literature that SMS interventions can reach hard-to-reach populations and be scaled for greater reach. Text messages are easily adapted, delivered in multiple languages, and accessed quickly and easily through the common use of cell phones (24).

Participants who received half or more of the exercise messages were more likely to meet PA guidelines, as supported by previous literature (7, 25, 26). Our results show a relatively short exposure of at least 12-weeks can influence self-reported PA. These findings align with prior literature and demonstrate SMS interventions effectiveness for PA can translate to rural settings (13, 27).

Evidence of effectiveness for SMS interventions in rural communities is important because SMS can be informed by theory and are evidence-based, dynamic, and adaptable. One promising approach involves dynamic feedback where messaging and goals change based on participant responses to prompts and questions (28).

We tested a combination of personalized and standard messages; however, did not design the study to test these programs separately. Currently, research is mixed on the added effects of personalized messages and what SMS designs, frequency, timing, and interactivity are most effective (15, 29). Almost no research exists exploring these questions for rural communities. Other research suggests these elements of SMS interventions may be important for engagement (30).

The multilevel *Heartland Moves* study required adaptation to the original protocol in response to the COVID-19 pandemic. The community and interpersonal level components had to be altered due to restrictions on in-person gatherings. As a result, the amount of control over, and intensity of, the community and interpersonal level interventions were impacted the most; however, we were able to encourage walking groups and PA through mailers and billboards.

Building on approaches in previous studies in these communities (e.g., relationship building) (8, 31), community-based partnerships were vital to the study team’s ability to recruit participants and pivot during the pandemic. The local community health coordinator (DD), who lived and worked in the region, was the key to building relationships with participants and community groups. When the pandemic began, the local coordinator assisted the research team in pivoting by gaining community input on the best ways to adapt the planned intervention. As part of this adaptation, the SMS messaging component of the intervention became a focal point of the PA promotion efforts. Highlighted in the results, more exercise messaging to participants was significantly associated with higher levels of total weekly PA. In a baseline analysis of the sample overall, we found occupational PA to be a key driver in meeting PA guidelines (22). SMS may effectively promote PA in rural communities, given messages are tailored to specific communities and consider the local context.

### 5.2 Limitations and strengths

There are several study limitations. The COVID-19 pandemic led to several adaptations to both the implementation of the intervention as well as the measurement and evaluation processes. Therefore, there was a reliance on self-report measures as accelerometer and GPS devices became challenging to administer, as well as a more limited scope of the intervention which may have impacted results. This includes a retention rate at follow-up of about 40%, which may present a bias though participants who did not participate in the project post baseline had statistically similar demographic characteristics. It is also important to note the effect of the COVID-19 pandemic on different domains of PA. Policies and practices relevant to accessing recreational opportunities and engaging in worksite activity may have hindered PA during this time. However, results also point to the sustainability of SMS as part of an intervention even during pandemic times. Even though we studied 14 rural communities, as with other settings, rural communities are diverse by socioeconomic status and race/ethnicity. Therefore, our findings cannot be generalized to all rural settings. Strengths of the current study include rigorous community engagement, which resulted in sustained community partnerships that have been beneficial for the communities in which we worked. This community-based and engaged work is vital to place-based health promotion efforts in community settings. This study also utilized a rigorous study design and analysis (i.e., community-based randomized controlled trial) targeting communities that tend to engage in lower PA. This study addressed a critical gap in research in rural communities that would benefit the most, even with the adaptations caused by the COVID-19 pandemic.

### 5.3 Future research

Our project and other recent studies highlight areas for future research. Much of the research on the effects of the built environment on PA has been conducted in urban and suburban settings (32). Additional research is needed on the influence of the physical environment (e.g., access to facilities, trails) in rural communities. Social network interventions are impactful in certain settings (e.g., schools) and more research is needed on social networks and PA promotion in rural communities (33).

Future research on SMS interventions in rural populations should distinguish the types of messaging (e.g., community-specific vs. exercise program, motivation vs. education-focused), timing (e.g., evening/morning, frequency), interactivity (reporting health behaviors, conducting real-time dialogue to identify PA cues, troubleshooting barriers to PA), presence (person-like qualities), and message design (language, tone, semantics, personalization) for effectiveness (15). Future research may benefit from co-designing SMS interventions with rural communities to address barriers to SMS intervention engagement and effectiveness (30).

SMS is cost-effective for some chronic disease conditions (e.g., diabetes, hypertension) (34); more scholarship is needed on the cost-effectiveness of SMS for promote 6bdcng PA, particularly in rural settings.

## 6 Conclusion

Walking trails are community resources offering accessibility to PA opportunities and can help to address disparities of chronic disease burden in rural areas. Efforts for PA promotion should involve community partnerships addressing the context of local communities. SMS may provide a sustainable approach to PA promotion but must be a part of a multifaceted approach. Future research should examine implementation efforts of the intervention at a larger scale in rural communities across the United States. It should examine different components of the current intervention for PA promotion across a range of rural communities.

## Data Availability

The raw data supporting the conclusions of this article will be made available by the authors, without undue reservation.
